# Clinical impact of combined assessment of myocardial inflammation and fibrosis using myocardial biopsy in patients with dilated cardiomyopathy: a multicentre, retrospective cohort study

**DOI:** 10.1136/openhrt-2025-003250

**Published:** 2025-03-12

**Authors:** Takafumi Nakayama, Keiko Ohta Ogo, Yasuo Sugano, Tetsuro Yokokawa, Hiromitsu Kanamori, Yoshihiko Ikeda, Michiaki Hiroe, Kinta Hatakeyama, Hatsue Ishibashi-Ueda, Kazufumi Nakamura, Kaoru Dohi, Toshihisa Anzai, Yoshihiro Seo, Kyoko Imanaka-Yoshida

**Affiliations:** 1 Department of Cardiology, Nagoya City University Graduate School of Medical Sciences, Nagoya, Japan; 2 Department of Pathology, National Cerebral and Cardiovascular Center, Suita, Japan; 3 Department of Cardiology, Keiyu Hospital, Yokohama, Japan; 4 Department of Cardiovascular Medicine, Fukushima Medical University, Fukushima, Japan; 5 Department of Cardiology, Gifu University Graduate School of Medicine, Gifu, Japan; 6 Department of Cardiology, National Center for Global Health and Medicine, Shinjuku-ku, Japan; 7 Department of Pathology, Hokusetsu General Hospital, Takatsuki, Japan; 8 Center for Advanced Heart Failure, Okayama University Hospital, Okayama, Japan; 9 Department of Cardiology and Nephrology, Mie University Graduate School of Medicine, Tsu, Japan; 10 Department of Cardiovascular Medicine, Hokkaido University Graduate School of Medicine, Sapporo, Japan; 11 Department of Cardiovascular Medicine, National Cerebral and Cardiovascular Center, Suita, Japan; 12 Department of Pathology and Matrix Biology, Mie University Graduate School of Medicine, Tsu, Japan

**Keywords:** Cardiomyopathy, Dilated, Inflammation, Myocarditis

## Abstract

**ABSTRACT:**

**Background:**

Among patients with dilated cardiomyopathy (DCM), myocardial inflammation and fibrosis are risk factors for poor clinical outcomes. Here, we investigated the combined prognostic value of these two factors, as evaluated using myocardial biopsy samples.

**Methods:**

This retrospective and multicentre study included patients with DCM—defined as LVEF of ≤45% and left diastolic diameter of >112% of predicted value, without evidence of secondary or ischaemic cardiomyopathy. In myocardial biopsy samples, inflammatory cells were counted using immunohistochemistry, and Masson’s Trichrome staining was performed to quantify the myocardial fibrosis as collagen area fraction (CAF). Higher myocardial inflammation was defined as leucocytes of ≥14/mm², including ≤4 monocytes/mm², with CD3^+^ T lymphocytes of≥7/mm². Greater myocardial fibrosis was defined as CAF of>5.9% by the Youden’s index. The primary endpoint was cardiac death or left ventricular assist device implantation.

**Results:**

A total of 255 DCM patients were enrolled (average age, 53.1 years; 78% males). Within this cohort, the mean LVEF was 28.0%, mean CAF was 10.7% and median CD3^+^ cell count was 8.3/mm^2^. During the median follow-up period of 2688 days, 46 patients met the primary endpoint. Multivariable Cox proportional hazard analyses revealed that CD3^+^ cell count and CAF were independent determinants of the primary endpoint. Kaplan–Meier analysis showed that patients with both higher myocardial inflammation and greater fibrosis had the worst prognosis (log-rank p<0.001). When myocardial inflammation was graded as one of three degrees: T lymphocytes of <13/mm² (low); 13 of 13.1–23.9/mm² (moderate); and T lymphocytes of ≥24 /mm² (high), patients with moderate inflammation exhibited a superior survival rate when CAF was ≤5.9%, but a worse survival rate when CAF was >5.9%.

**Conclusions:**

Having both biopsy-proven higher myocardial inflammation and greater fibrosis predicted the worst clinical prognosis in patients with DCM.

**WHAT IS ALREADY KNOWN ON THIS TOPIC**

- Myocardial inflammatory cell infiltration and high left ventricular fibrosis are established predictors of poor prognosis in patients with dilated cardiomyopathy (DCM). However, their combined prognostic impact remains poorly understood.

**WHAT THIS STUDY ADDS**

- This multicentre cohort study demonstrated that both the degree of inflammatory cell infiltration and the extent of fibrosis in myocardial biopsy specimens independently predict adverse outcomes in DCM. Notably, the coexistence of severe inflammation and extensive fibrosis identified patients at highest risk for poor clinical outcomes.

**HOW THIS STUDY MIGHT AFFECT RESEARCH, PRACTICE OR POLICY**

- These findings advocate for close monitoring and careful consideration of therapeutic strategies in DCM patients with concurrent high myocardial inflammation and fibrosis. Furthermore, this high-risk population represents an important target cohort for future clinical trials evaluating novel immunosuppressive strategies.

## Introduction

 Dilated cardiomyopathy (DCM) is defined as left ventricular (LV) dilatation and systolic dysfunction, which are not explained by abnormal loading conditions.^1^ Patients with DCM experience degradation of the quality of life and prognosis due to heart failure symptoms and arrhythmia. The underlying substrates vary and comprise both genetic and environmental factors.^2^,^4^

It has recently been elucidated that chronic myocardial and surrounding inflammation are associated with poor clinical outcomes in DCM.^5^,^7^ This condition is termed inflammatory DCM and can be diagnosed by endomyocardial biopsy.^8 9^ In two previous studies, we assessed the relationship between biopsy-proven myocardial inflammation and prognosis, which revealed poor clinical outcomes among patients with DCM who met the myocardial inflammation criteria defined by the European Society of Cardiology (ESC).^5 6^ Furthermore, we found that three-tiered risk stratification according to the infiltrating CD3^+^ lymphocyte count was useful for predicting the detailed prognosis in DCM patients (INDICATE study).^5^ Notably, myocardial fibrosis is also a well-known cause of LV remodelling and a classical risk factor for poor clinical outcomes.^10^ While the predictive value of myocardial fibrosis is often assessed by cardiac magnetic resonance imaging (CMRI), it can also be evaluated using endomyocardial biopsy.^6^ To date, no study has investigated the prognostic impact of the combined simultaneous evaluation of myocardial inflammation and fibrosis in DCM patients.

In the current study, we aimed to investigate the combined prognostic value of myocardial fibrosis and myocardial inflammation, both evaluated using endocardial biopsy, in patients with DCM.

## Methods

### Study population and protocol

We performed a multicentre, retrospective, observational study and a sub-analysis of previously published research.^5^ The study included patients with DCM who underwent myocardial biopsy between January 2004 and December 2014, enabling an over 5 year follow-up period. From patients’ medical records, we obtained their clinical history, physical findings, echocardiographic parameters and haemodynamic data. LV dysfunction suspicious of DCM was defined as LVEF of ≤45% and LV diastolic diameter of >112% of the predicted value.^5^ The predicted value was calculated using the following formula: 45.3 × (body surface area)^1/3^ − (0.03×Age) − 7.2.^11^ LVEF was calculated using the Bi-plane modified-Simpson method or with the Teichholz method in 80 patients who lacked LVEF using the Bi-plane modified-Simpson method. DCM was comprehensively diagnosed by the attending physician based on clinical examinations, including endomyocardial biopsy. Although DCM is sometimes diagnosed after confirming the absence of myocardial inflammation, here we used the term DCM in a broad sense, excluding secondary cardiomyopathies.

Patients were excluded if they exhibited coronary stenosis of >50% at the main branch; severe primary valvular disease; history of uncontrollable or untreated hypertension for ≥1 year before LV dysfunction was documented; and other secondary cardiomyopathies, such as sarcoidosis or amyloidosis. We also excluded patients with a history of malignant disease, cardiac surgery, acute myocarditis and collagen disease; patients receiving current or prior immunosuppressive therapy; and patients with acute infection on the day of the biopsy. The clinical primary endpoint was defined as a composite: cardiovascular death or LV assist device implantation.

This retrospective study was approved by the Institutional Review Board of the National Cerebral and Cardiovascular Centre (M27-063), Nagoya City University (60-16-0086) and other institutes. It was conducted in accordance with the principles of the Declaration of Helsinki.

### Process for preparing histopathological samples

The patients’ biopsy samples were stained with haematoxylin–eosin, Masson’s trichrome and immunohistochemistry for inflammatory cells, using an autostainer at the National Cerebral and Cardiovascular Centre. T lymphocytes and macrophages were identified using anti-CD3 antibody and anti-CD68 antibody, respectively. Detailed products and methods are presented in online supplemental table S1.

### Histopathological assessment for myocardial inflammation

Whole slides were digitally scanned at the National Cerebral Cardiovascular Centre. Microscopic images were randomly taken of five high-power fields on each slide, and then, two blinded pathologists (KO-O and HI-U) counted the CD3^+^ cells and CD68^+^ cells, excluding cells in vessels^5 6^ (online supplemental figure S1A–C). First, we assessed the presence of myocardial inflammation using the ESC criteria (≥ 14 leucocyte/mm², including ≤4 monocytes/mm² and CD3^+^ T lymphocytes≥7 /mm²).^9^ Second, we divided the patients into three groups according to the INDICATE study criteria, as follows: T lymphocytes of <13/mm² (low); 13.1–23.9/mm² (moderate); and T lymphocytes of ≥24/mm² (high).

### Histopathological assessment of fibrotic area

Myocardial fibrosis was evaluated using Masson’s trichrome-stained specimens. The collagen area fraction (CAF) was calculated as the ratio of the blue-colour area (excluding the endocardium) to the area of the whole specimen. We quantified the blue area as fibrosis using the Positive Pixel Count algorithm version 9 of Aperio ImageScope^5^ (online supplemental figure S1D,E). The cut-off value of CAF for the primary endpoint was determined using Youden’s index, derived from the receiver operating characteristics curve. We calculated intraclass correlation coefficients (ICCs) to evaluate the intra- and inter-rater reliabilities for CAF, as determined by TN and KO-O among the latest 30 cases at the National Cerebral and Cardiovascular Centre. An ICC of ≥0.8 was considered the preferred reliability level.^12^

### Statistical analysis

Continuous values were expressed as the mean±SD when normally distributed and the median (IQR) when not normally distributed. Average normally distributed continuous values were compared between groups using the Student’s t-test, whereas the Mann–Whitney U-test was used for values that were not normally distributed. Categorical values were expressed as number (percentage) and compared using the X^2^ test.

Cox proportional hazard analysis was performed to identify factors associated with the primary endpoint. We selected the parameters based on a previous meta-analysis investigating risk factors in heart failure.^13^ Since our retrospective study did not include data regarding systolic blood pressure, we used a history of hypertension instead. Additionally, we excluded the duration of heart failure from our multivariable analysis due to the lack of information for 82 patients. Variables with a *P* value of <0.05 in univariable analysis were entered into multivariable analyses. Regarding the incidence of the primary endpoint, multivariable Cox proportional hazard analyses included the infiltrating T-lymphocyte count, CAF and parameters from each category, such that the analyses were limited to include no more than four variables at once. We selected the number of CD3^+^ cells for grading the myocardial inflammation in the multivariable analyses since the ESC statement designates lymphocytes as the main diagnostic criterion, while considering macrophages/monocytes as a supportive finding.^9^ The log–log plot of the event-free rate was used to test the Cox proportional hazard assumption. Kaplan–Meier curves were drawn to compare event-free survival rates between groups, and the significance was assessed using the log-rank test. The patient’s follow-up data were collected until the loss of follow-up or the primary outcomes. The c-statistics were used to assess the superiority of the combined diagnostic performance of myocardial inflammation and fibrosis compared with using myocardial inflammation or fibrosis alone for the primary endpoint. The univariable and multivariable logistic analyses were performed to identify factors associated with the infiltrating T lymphocytes and CAF.

All statistical analyses were performed using SPSS software ver. 26 (IBM Corp., Armonk, NY, USA), and two-sided *P* values of <0.05 were considered significant.

### Patient and public involvement

Patients or the public were not involved in the design, conduct, reporting or dissemination of our research.

## Results

### Baseline characteristics

Among all 265 DCM patients registered from eight institutions, three were excluded due to the absence of cardiomyocytes, one due to the presence of amyloid deposits and six due to the absence of Masson–Trichrome staining (online supplemental figure S2). Finally, 255 patients were included in our analyses. Table 1 presents the baseline characteristics of all patients. The average age was 53.1 years, 78% were male and the mean LVEF was 28.0%. Histograms of CD3^+^ cells, CD68^+^ cells and CAF are presented in online supplemental figure S3. For quantifying CAF, the ICCs of intra- and inter-rater reliabilities were 0.995 (95% CI: 0.990 to 0.998) and 0.98 (95% CI: 0.95 to 0.99).

**Table 1:** Baseline characteristics

	All patients	Event free	Event occurrence	P value
	n=255	n=209	n=46	
Demographics				
Age, year	53.1±15.1	53.4±14.5	50.0±17.4	0.13
Male, n (%)	198 (78%)	158 (76%)	40 (87%)	0.094
Body mass index, kg/m^2^	23.7±4.4	23.9±4.5	22.6±3.8	0.081
NYHA class III or IV, n (%)	64 (25%)	40 (19%)	24 (52%)	< 0.001
Duration of heart failure, month (n=173)	4.0 (1.0–22.5)	3.0 (1.0–8.8)	50.0 (6.0–73.5)	< 0.001
Hypertension, n (%)	100 (39%)	92 (44%)	8 (17%)	0.001
Dyslipidaemia, n (%)	114 (45%)	98 (47%)	16 (35%)	0.135
Diabetes mellitus, n (%)	32 (13%)	26 (12%)	6 (13%)	0.91
Atrial fibrillation, n (%)	57 (22%)	52 (25%)	11 (24%)	0.89
Smoking history (n=233)	87 (37%)	74 (35%)	13 (28%)	0.57
Chronic obstructive pulmonary disease	5 (2%)	5 (2%)	0 (0%)	0.29
Ventricular tachycardia (non-sustained and sustained), n (%)	69 (27%)	44 (21%)	25 (54%)	< 0.001
Laboratory measurements				
BNP, pg/mL	191 (90–494)	168 (76–407)	416 (217–1025)	< 0.001
Estimated GFR, mL/min/1,73 m2	70.4±25.1	70.1±23.6	71.6±31.4	0.70
White blood cells, /µL (n=254)	6310±1837	6287±1877	6415±1659	0.67
Neutrophils, /µL (n=228)	3798±1510	3761±1561	3959±1271	0.44
Lymphocytes, /µL (n=228)	1829±650	1869±709	1652±709	0.47
C-reactive protein, mg/dL	0.13 (0.05–0.34)	0.12 (0.04–0.31)	0.21 (0.08–0.54)	0.011
Echocardiography				
LVEDD, mm	65.8±8.2	65.2±7.6	68.9±10.1	0.005
LVEF, %	28.0±8.6	28.7±8.3	24.6±9.1	0.003
Haemodynamics				
PCWP, mmHg (n=244)	12.8±8.5	12.0±8.1	16.5±9.7	0.001
Cardiac index, L/min/m^2^ (n=220)	2.5±1.1	2.5±0.6	2.5±2.2	0.95
Medication at discharge				
β-blocker, n (%)	231 (91%)	188 (90%)	43 (93%)	0.46
ACE inhibitor or ARB, n (%)	220 (86%)	184 (88%)	36 (78%)	0.081
MRA, n (%)	107 (42%)	86 (41%)	21 (46%)	0.58
Loop diuretics, n (%)	180 (71%)	145 (69%)	35 (76%)	0.37
Amiodarone, n (%)	42 (16%)	30 (14%)	12 (26%)	0.052
Anticoagulationn, n (%)	135 (53%)	126 (60%)	32 (70%)	0.24
Statin, n (%)	52 (20%)	43 (21%)	9 (20%)	0.88
Pathological findings				
CD3, cells/mm^2^	8.3 (4.0–16.0)	8.0 (4.0–14.0)	14.0 (6.0–20.4)	0.007
CD68, cells/mm^2^	26.0 (14.0–38.0)	25.1 (12.1–38.0)	28.2 (19.7–43.0)	0.076
Collagen area fraction, %	10.7±8.4	10.2±8.5	12.6±7.8	0.087

The patients numbers for white blood cells, and neutrophils and lymphocytes values: n=208 and 46, 185 and 43 in the event -free group and event group, respectively. Those for PCWP and cardiac index values: n=199 and 45, 180 and 40 in the event -free group and event group, respectively.

ACE, angiotensin-converting enzyme; ARB, angiotensin receptor blocker; BNP, brain natriuretic peptide; GFR, glomerular filtration ratio; LVEDD, left ventricular end-diastolic diameter; LVEF, left ventricular ejection fraction; MRA, mineralocorticoid receptor antagonistNYHA, New York Heart Association; PCWP, pulmonary capillary wedged pressure

During the median observational period of 2688 days (IQR, 1448–3633 days), 46 patients met the primary endpoints. The CAF cut-off value was determined to be 5.9% (online supplemental figure S4).

### Pathohistological findings and patient prognosis

Univariable Cox proportional hazard analyses revealed 10 variables as significant for the primary endpoint (table 2). The infiltrating CD3^+^ cell count and CAF were independent predictors of the primary endpoint in multivariable Cox proportional hazard analyses, including each category of parameters (table 3). The Cox proportional hazard assumption was verified to be met by confirming parallel curves and the absence of interactions among all variables in the multivariable analyses.

**Table 2:** Cox proportional hazard analyses for the primary endpoint

	Univariable	
	HR	P value
Demographics		
Age, per 10 year	0.88 (0.72–1.08)	0.22
Mal, vs female	0.56 (0.24–1.31)	0.18
Body mass index, kg/m^2^	0.93 (0.86–1.00)	0.051
NYHA class	2.06 (1.54–2.76)	< 0.001
Duration of heart failure, per 6 month (n=173)	1.06 (1.03–1.09)	< 0.001
Hypertension	0.33 (0.15–0.70)	0.004
Dyslipidaemia	0.61 (0.33–1.12)	0.11
Diabetes mellitus	1.08 (0.46–2.54)	0.87
Atrial fibrillation	1.04 (0.52–2.11)	0.91
Smoking history (n=233)	0.88 (0.45–1.71)	0.70
Chronic obstructive pulmonary disease	0.05 (0.00–841.1)	0.54
Laboratory measurements		
Log BNP	4.83 (2.65–8.80)	< 0.001
Estimated GFR, per 10 mL/min/1,73 m2	1.01 (0.89–1.14)	0.94
C-reactive protein, mg/dL	1.23 (0.90–1.69)	0.19
Echocardiography		
LVEDD, pe -5mm	1.22 (1.03–1.44)	0.020
LVEF, pe -10%	0.62 (0.43–0.88)	0.007
Haemodynamics		
PCWP, mmHg	1.06 (1.03–1.09)	< 0.001
Cardiac index, L/min/m^2^	1.01 (0.77–1.31)	0.97
Medication at discharge		
β-blocker, n	1.55 (0.48–5.01)	0.46
ACE inhibitor or ARB, n	0.57 (0.28–1.14)	0.11
Pathological findings		
CD3^+^ cells, per 5 /mm2	1.10 (1.05–1.16)	< 0.001
CD68 cells, per 5 /mm2	1.07 (1.02–1.13)	0.009
Collagen area fraction, %	1.04 (1.01–1.06)	0.010

ACE, angiotensin-converting enzyme; ARB, angiotensin receptor blockerBNP, brain natriuretic peptide; GFR, glomerular filtrating ratio; LVEDD, left ventricular end-diastolic diameter; LVEF, left ventricular ejection fraction; NYHA, New York Heart Association; PCWP, pulmonary capillary wedged pressure

**Table 3:** Cox proportional hazard analyses for the primary endpoint

	Univariable		Multivariable	
	HR	P value	HR	P value
Demographics				
NYHA class	2.06 (1.54–2.76)	< 0.001	1.83 (1.35–2.48)	< 0.001
Hypertension	0.33 (0.15–0.70)	0.004	0.36 (0.16–0.77)	0.009
Pathological findings				
CD3^+^ cells, per 5/mm^2^	1.10 (1.05–1.16)	< 0.001	1.07 (1.00–1.13)	0.043
Collagen area fraction, %	1.04 (1.01–1.06)	0.010	1.04 (1.01–1.07)	0.010
Laboratory measurements				
Log BNP	4.83 (2.65–8.80)	< 0.001	4.98 (2.65–9.35)	< 0.001
Pathological findings				
CD3^+^ cells, per 5/mm^2^	1.10 (1.05–1.16)	< 0.001	1.09 (1.03–1.16)	0.002
Collagen area fraction, %	1.04 (1.01–1.06)	0.010	1.03 (1.00–1.06)	0.035
Echocardiography				
LVEDD, pe -5mm	1.22 (1.03–1.44)	0.020	1.14 (0.95–1.38)	0.17
LVEF, pe -10%	0.62 (0.43–0.88)	0.007	0.67 (0.45–0.99)	0.045
Pathological findings				
CD3^+^ cells, per 5/mm^2^	1.10 (1.05–1.16)	< 0.001	1.09 (1.04–1.15)	0.001
Collagen area fraction, %	1.04 (1.01–1.06)	0.010	1.04 (1.01–1.07)	0.006
Haemodynamics				
PCWP, mmHg	1.06 (1.03–1.09)	< 0.001	1.05 (1.02–1.09)	< 0.001
Pathological findings				
CD3^+^ cells, per 5/mm^2^	1.10 (1.05–1.16)	< 0.001	1.10 (1.04–1.17)	0.001
Collagen area fraction, %	1.04 (1.01–1.06)	0.010	1.03 (1.00–1.06)	0.001

BNPbrain natriuretic peptideLVEDDleft ventricular end-diastolic diameterLVEFleft ventricular ejection fractionNYHANew York Heart AssociationPCWPpulmonary capillary wedged pressure

**Figure 1:**
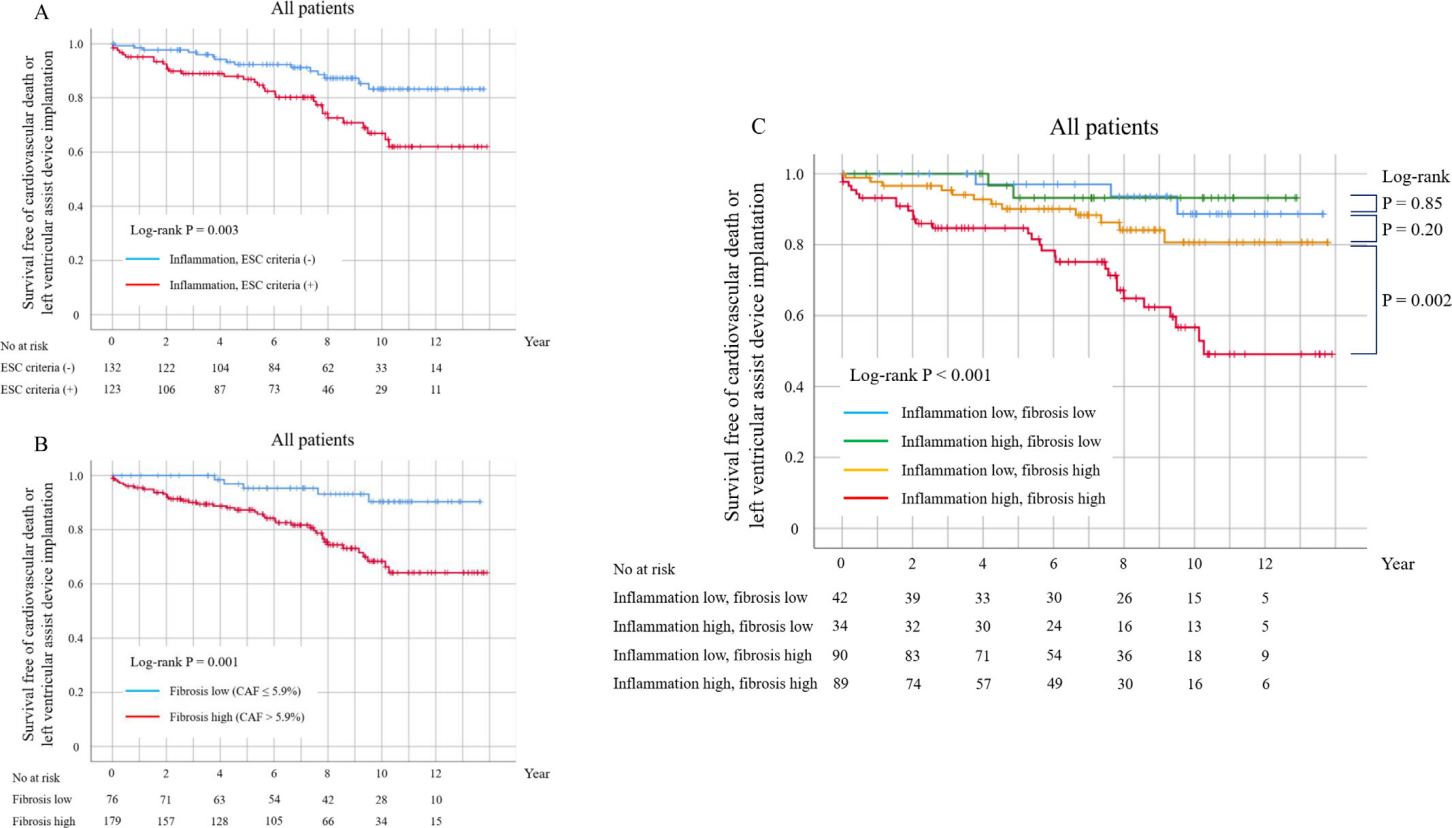
Kaplan–Meier curves for the primary endpoint based on myocardial inflammation according to ESC criteria (**A**), myocardial fibrosis with a 5.9% collagen area fraction (CAF) as a cut-off (**B**), and the simultaneous presence of these two factors (**C**). Patients with myocardial inflammation defined by ESC criteria or with CAF of >5.9% had worse outcomes than patients without either. Patients with the presence of both factors had the worst outcome compared with the other groups. ESC, European Society of Cardiology.

**Figure 2:**
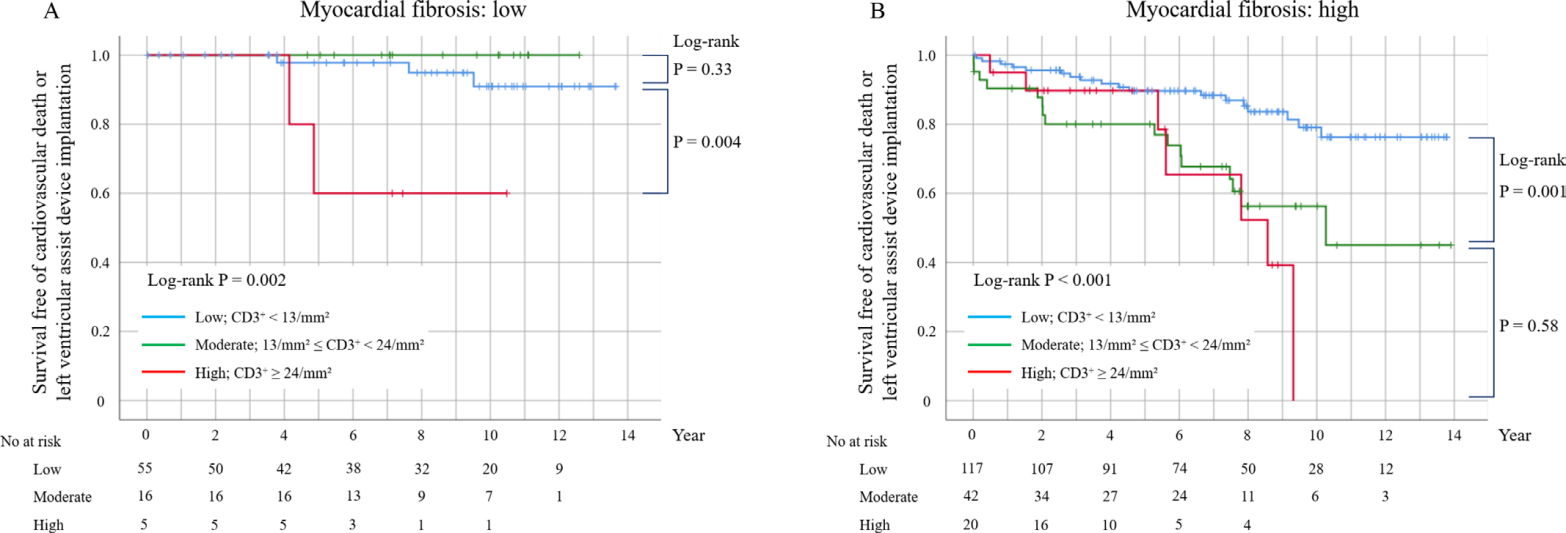
Kaplan–Meier curves for the primary endpoint according to the INDICATE criteria. Patients with moderate myocardial inflammation exhibited a significantly superior survival rate when also having low myocardial fibrosis (**A**) and a significantly poorer prognosis when also having high myocardial fibrosis (**B**).

The Kaplan–Meier curves showed significantly lower survival rates among patients with higher myocardial inflammation (ESC criteria) and patients with greater myocardial fibrosis (CAF, >5.9%), compared with those with lower myocardial inflammation and fibrosis (figure 1A,B). Furthermore, patients with both higher myocardial inflammation and greater fibrosis had the worst outcomes compared with the other groups (log-rank p<0.001, figure 1C). Using the CD3^+^ cell cut-off value defined in the INDICATE study, patients with moderate CD3^+^ cells (13–24/mm^2^) exhibited a significantly superior survival rate when also showing lower myocardial fibrosis (CAF, ≤5.9%), and a significantly inferior survival rate when also exhibiting higher myocardial fibrosis (CAF, >5.9%) (figure 2 and online supplemental figure S5). The c-statistics analysis revealed a higher area under the curve for the combined assessment of myocardial CD3^+^ cell count and CAF (0.69) compared with either CD3^+^ cells (0.63) or CAF (0.63) alone for the primary endpoint (online supplemental figure S6).

### Factors associated with myocardial inflammation and fibrosis

Baseline characteristics, according to high or low myocardial inflammation (ESC criteria) and fibrosis (CAF, >5.9%), are summarised in table 4. The results of logistic regression analyses are presented in online supplemental tables S2 and S3. Univariable logistic analyses revealed that positivity for ESC criteria myocardial inflammation and CAF of >5.9% were not associated with each other. Linear regression analysis also showed no significant relationship between the CD3^+^ cell count and CAF (online supplemental figure S7). Multivariable logistic analyses identified that myocardial inflammation was independently associated with age and NYHA class and that only a history of hypertension was independently associated with myocardial fibrosis (online supplemental tables S2 and S3).

**Table 4:** Baseline characteristics

	Inflammation (+)	Inflammation (-)	P value	Fibrosis (+)	Fibrosis (-)	P value
	n=123	n=132		n=179	n=76	
Demographics						
Age, year	50.8±15.4	55.3±14.5	0.018	52.6±15.3	54.2±14.5	0.44
Male, n (%)	91 (74%)	32 (24%)	0.18	141 (79%)	57 (75%)	0.51
Body mass index, kg/m^2^	23.7±3.9	23.7±4.8	0.99	23.4±4.5	24.3±4.1	0.14
NYHA class III or IV, n (%)	40 (33%)	24 (18%)	0.008	44 (25%)	20 (26%)	0.77
Hypertension, n (%)	43 (35%)	57 (43%)	0.18	61 (34%)	39 (51%)	0.010
Dyslipidaemia, n (%)	48 (39%)	66 (50%)	0.078	78 (44%)	36 (47%)	0.58
Diabetes mellitus, n (%)	14 (11%)	18 (14%)	0.59	22 (12%)	10 (13%)	0.85
Atrial fibrillation, n (%)	26 (21%)	31 (23%)	0.65	41 (23%)	16 (21%)	0.75
Ventricular tachycardia (non-sustained and sustained), n (%)	29 (24%)	40 (30%)	0.23	53 (30%)	16 (21%)	0.16
Laboratory measurements						
BNP, pg/mL	237 (91–546)	188 (82–404)	0.29	254 (105–546)	132 (53–306)	0.002
Estimated GFR, mL/min/1,73 m2	72.4±26.2	68.4±24.0	0.21	71.5±24.7	67.6±26.0	0.26
C-reactive protein, mg/dL	0.15 (0.06–0.30)	0.11 (0.04–0.34)	0.55	0.15 (0.06–0.34)	0.08 (0.03–0.26)	0.020
White blood cells, /µL (n=254)	6346±1618	6276±2027	0.76	6333±1774	6258±1989	0.77
Neutrophils, /µL (n=228)	3702±1239	3886±1722	0.36	3847±1495	3680±1552	0.45
Lymphocytes, /µL (n=228)	1882±683	1780±616	0.24	1802±656	1894±633	0.34
Echocardiography						
LVEDD, mm	65.7±8.0	65.9±8.5	0.86	65.7±8.7	66.2±7.2	0.69
LVEF, %	27.2±8.8	28.7±8.3	0.17	27.6±8.8	28.7±8.0	0.36
Haemodynamics						
PCWP, mmHg	13.8±9.1	11.9±7.9	0.093	13.6±8.4	11.1±8.7	0.034
Cardiac index, L/min/m^2^	2.50±0.67	2.51±1.37	0.91	2.44±0.58	2.66±1.83	0.19
Medication at discharge						
β-blocker, n (%)	109 (89%)	122 (92%)	0.30	163 (91%)	68 (89%)	0.69
ACEI or ARB	102 (83%)	118 (89%)	0.13	156 (87%)	64 (84%)	0.53
MRA	55 (45%)	52 (39%)	0.39	78 (44%)	29 (38%)	0.42
Pathological findings						
CD3 cells, /mm^2^	16.0 (12.0–21.1)	4.3 (2.0–7.5)	< 0.001	10.0 (4.0–16.2)	8.0 (4.1–14.0)	0.52
CD68 cells, /mm^2^	32.0 (21.8–48.0)	20.0 (8.4–34.2)	< 0.001	26.1 (14.0–38.0)	24.0 (12.7–43.7)	0.67
Collagen area fraction, %	11.3±8.4	10.0±8.4	0.23	13.3±8.7	4.3±1.9	< 0.001

ACE, angiotensin-converting enzyme; ARB, angiotensin receptor blocker; BNP, brain natriuretic peptide; GFR, glomerular filtrating ratio; LVEDD, left ventricular end-diastolic diameter; LVEF, left ventricular ejection fraction; MRA, mineralocorticoid receptor antagonistNYHA, New York Heart Association; PCWP, pulmonary capillary wedged pressure

## Discussion

The current study is the first to investigate the combined prognostic impact of pathological myocardial inflammation and fibrosis in patients with DCM. This retrospective study included the greatest enrolment among investigations where myocardial biopsy has been performed while alive and its prognostic utility assessed in patients with DCM. Our results demonstrated that DCM patients with higher myocardial inflammation and greater myocardial fibrosis had poorer outcomes compared with other groups. Furthermore, among patients with infiltrating T-lymphocyte counts ranging between 13/mm^2^ and 24/mm^2^ (moderate in the INDICATE criteria), prognosis depended on the extent of myocardial fibrosis.

### Myocardial inflammation in patients with DCM

Over recent decades, growing evidence has revealed the clinical significance of infiltrative inflammatory cells in patients with DCM. The concept of ‘inflammatory DCM’ was proposed by Maisch et al,^14^ suggesting that DCM patients be divided according to the presence of myocardial inflammation and virus using biopsy samples. In the ESC guidelines, pathological myocardial inflammation is defined by myocardial infiltrative leucocytes of ≥14/mm^2^, which can include up to 4/mm^2^ of macrophages.^9^ Using these criteria, we previously demonstrated a worse prognosis among patients with biopsy-proven inflammatory DCM, compared with those without.^5 6^ Clinical prognosis is also adversely affected by biopsy-proven myocardial inflammation in other cardiac diseases, including cardiac amyloidosis,^15^ Fabry disease,^16^ hypertrophic cardiomyopathy (HCM)^17^ and arrhythmogenic right ventricular cardiomyopathy.^18^ The cut-off values for myocardial infiltrative T lymphocytes were similar among these different diseases, suggesting that over 10–14/mm^2^ of infiltrative T lymphocytes is an adverse prognostic indicator in cardiac disease in general.

Although prednisone therapy does not yield clinical benefit for all DCM patients,^19^ Wojnicz *et al* demonstrated that the use of prednisone and azathioprine improved cardiac functions and symptoms among patients with HLA upregulation.^20^ Moreover, Frustaci *et al* showed that immunosuppressive therapy with prednisone and azathioprine improved cardiac function in DCM patients with myocardial inflammation (>7/mm^2^ of CD3-positive lymphocytes or >14/mm^2^ of leukocytes).^21^ These findings indicate that immunosuppressive therapy can be an effective treatment in carefully selected patients with DCM and myocardial infiltrative inflammatory cells.

The cause of myocardial inflammation in cardiomyopathy is not fully understood, but infection and autoimmunity are classically considered likely explanations.^2 14^ Logistic regression analysis (online supplemental table S2) revealed only age and New York Heart Association class as independent determinants. Moreover, our findings reconfirmed heterogeneous backgrounds for myocardial inflammation, with no other identified associated factors, including circulating white blood cells and lymphocytes. We additionally investigated the circulating white blood cells, neutrophils and lymphocytes in the Cox proportional hazard analyses (online supplemental table S4). Interestingly, the decrease in circulating lymphocytes was a significant variable in the univariable analysis. However, it turned insignificant in the multivariable analysis, whereas the myocardial infiltrating CD3^+^ cells and fibrosis stayed significant. Based on these results, we believe the myocardial inflammation was not totally dependent on systemic inflammation. Importantly, regardless of the cause, we can recognise that a high myocardial infiltrating inflammatory cell count is an indicator of worse clinical condition.

### Myocardial fibrosis in patients with DCM

The extent of myocardial fibrosis is well-known to be associated with poor clinical outcomes in patients with DCM. Fibrosis is often assessed by CMRI with late gadolinium enhancement (LGE), and meta-analysis has revealed that LGE presence has a prognostic impact on DCM patients.^10^ However, LGE reflects myocardial inflammation and oedema as well as myocardial fibrosis^22^ and is considered unsuitable for quantifying interstitial-myocardial fibrosis.^23 24^ CMRI with extracellular volume (ECV) was recently found to show prognostic utility in patients with DCM and negative LGE.^25^ On the other hand, a previous retrospective investigation demonstrated that higher LGE predicted poor outcomes, while endomyocardial biopsy did not in the same cohort,^26^ suggesting that LGE and biopsy-proven fibrosis differed in their reflected content and area. Only biopsy enables direct documentation of myocardial fibrosis.

Notably, there is not yet evidence that biopsy-proven CAF has major predictive ability in DCM. Studies have demonstrated both that biopsy-proven fibrosis predicted prognosis and that it did not.^6 26^ Although right ventricular endomyocardial biopsy can predict LV fibrosis,^27^ sampling error may be a problem in endomyocardial biopsy in a small-sized study.

We found low specificity when using a CAF cut-off value of 5.9%; therefore, we verified another cut-off value of 8.8% (sensitivity: 0.63, specificity: 0.55), which was determined based on the nearest point from the points with a sensitivity of 1.0 and specificity of 1.0 (online supplemental figure S8). When using this cut-off value of 8.8%, we found that the main results did not change (online supplemental figure S9). Thus, in the current study, we chose to apply 5.9% as the CAF cut-off value, which had high sensitivity and might indicate the normal range of CAF (online supplemental figure S10).

In our logistic analysis, only history of hypertension was independently and negatively associated with CAF of >5.9% (online supplemental table S3). Since history of hypertension also served as an alternative parameter for blood pressure in our study, this result likely reflects that maintained blood pressure was associated with better clinical prognosis. Our logistic analysis revealed no other causes, supporting the heterogeneous background in myocardial fibrosis. Notably, myocardial inflammation is also a substrate for myocardial fibrosis.^28 29^ Previous research shows that the myocardial infiltrative M2 macrophage count is significantly associated with the CAF.^6^ There remains a need for further exploration of the role of M2 macrophages in establishing myocardial fibrosis.

### Simultaneous presence of myocardial inflammation and fibrosis

The baseline parameters that significantly differed between patients with and without myocardial inflammation were not the same parameters that differed between patients with and without fibrosis (table 4). Furthermore, in the Cox proportional hazard analyses, both factors were independently associated with the primary endpoint. Thus, myocardial fibrosis and inflammation did not seem to be induced by the same mechanism. Notably, even without knowing the primary cause of infiltrating T lymphocytes and myocardial fibrosis, we have demonstrated their significant utility for predicting prognosis in DCM. Similar to our current results, we previously revealed that HCM patients with both biopsy-proven myocardial inflammation and fibrosis had the worst prognosis compared with other patient subsets.^17^ We also investigated the correlation between myocardial inflammation and fibrosis and LV remodelling. At the echocardiographic follow-up after 6–12 months, we observed significantly lower improvement in LV diastolic diameter, LV systolic diameter and LV ejection fraction among patients with both myocardial inflammation according to ESC criteria and CAF of >5.9%, compared with other patients (online supplemental table S5).

By dividing DCM patients into three levels of inflammation according to the INDICATE criteria, we demonstrated poor clinical prognoses among the patients with high myocardial inflammation (CD3^+^ T lymphocytes, ≥24/mm^2^), and the patients with moderate myocardial inflammation (13–23.9/mm^2^) and higher myocardial fibrosis (CAF, >5.9%). Based on this, we propose that patients with moderate myocardial inflammation and CAF of >5.9%, as well as patients with high inflammation, should be considered to have insufficient reserve capacity for 10 years with the current guideline-based medicine.

A history of hypertension was a strong indicator of the primary endpoint, suggesting a need to further investigate the association of hypertensive heart diseases. We performed the Cox proportional hazard analyses among only the 77 patients with systolic blood pressure data (online supplemental table S6) and confirmed that blood pressure was independently related to the primary endpoint. However, the history of hypertension became non-significant in multivariable analysis. These results did not change when the CD3^+^ cell count and CAF were entered into the analysis. Therefore, we believe that hypertension history indicated that blood pressure was being well-maintained, rather than being associated with hypertension itself or hypertensive heart disease.

### Clinical implications

Based on our findings, we recommend providing close monitoring or careful consideration to ensure the most optimal current therapy for two groups of patients. One group is the patients who meet the myocardial inflammatory condition, according to ESC criteria or a T-lymphocyte count ranging from 13/mm² to 24/mm², and have a CAF of >5.9%. The other group is the patients with high myocardial inflammation according to the INDICATE criteria, with a T-lymphocyte count of 24/mm² or higher, regardless of CAF levels. Quantification of CD3^+^ and CD68^+^ cells and CAF is available at almost every institute. We recommend that these quantifications should be included in biopsy reports. Furthermore, further research investigating the efficacy of immunosuppressive therapy among DCM patients may help improve the treatment for the above-listed patient groups.

### Study limitations

The current study had several limitations. First, it was retrospective in design, and thus further prospective research is needed to confirm our results. Second, the systolic blood pressure data were missing for the majority of patients. Third, we could not exclude the influence of biopsy sampling error on our results. To minimise the effects of sampling error, we evaluated all samples of specimens. Fourth, this study did not clarify the primary causes of inflammation and fibrosis. Information was not available regarding viral genome status, circulating autoantibodies and gene mutation for cardiomyopathy. The data on circulating cytokine levels were also unavailable, which could provide possible mechanisms for the inflammation. Notably, these examinations are not covered by insurance in Japan, and it was difficult to additionally investigate them in this retrospective multicentre analysis. Further basic research is warranted to deepen our knowledge of the mechanisms of inflammation and fibrosis.

## Conclusion

Higher myocardial infiltrating inflammatory cell count and greater CAF, evaluated using biopsy samples, predicted worse prognosis in patients with DCM. Patients with both high myocardial inflammation and fibrosis had the worst clinical outcomes. We recommend careful clinical follow-up for these patients.

## supplementary material

10.1136/openhrt-2025-003250 online supplemental figure 1

10.1136/openhrt-2025-003250 online supplemental figure 2

10.1136/openhrt-2025-003250 online supplemental figure 3

10.1136/openhrt-2025-003250 online supplemental figure 4

10.1136/openhrt-2025-003250 online supplemental figure 5

10.1136/openhrt-2025-003250 online supplemental figure 6

10.1136/openhrt-2025-003250 online supplemental figure 7

10.1136/openhrt-2025-003250 online supplemental figure 8

10.1136/openhrt-2025-003250 online supplemental figure 9

10.1136/openhrt-2025-003250 online supplemental figure 10

10.1136/openhrt-2025-003250 online supplemental table 1

## Data Availability

Data are available upon reasonable request.
