# Supplementary figures and images for: Clinical impact of combined assessment of myocardial inflammation and fibrosis using myocardial biopsy in patients with dilated cardiomyopathy: a multicentre, retrospective cohort study
Source: Open Heart. 2025 Mar 12;12(1):e003250. doi: 10.1136/openhrt-2025-003250 (PMC11907087; doi:10.1136/openhrt-2025-003250)

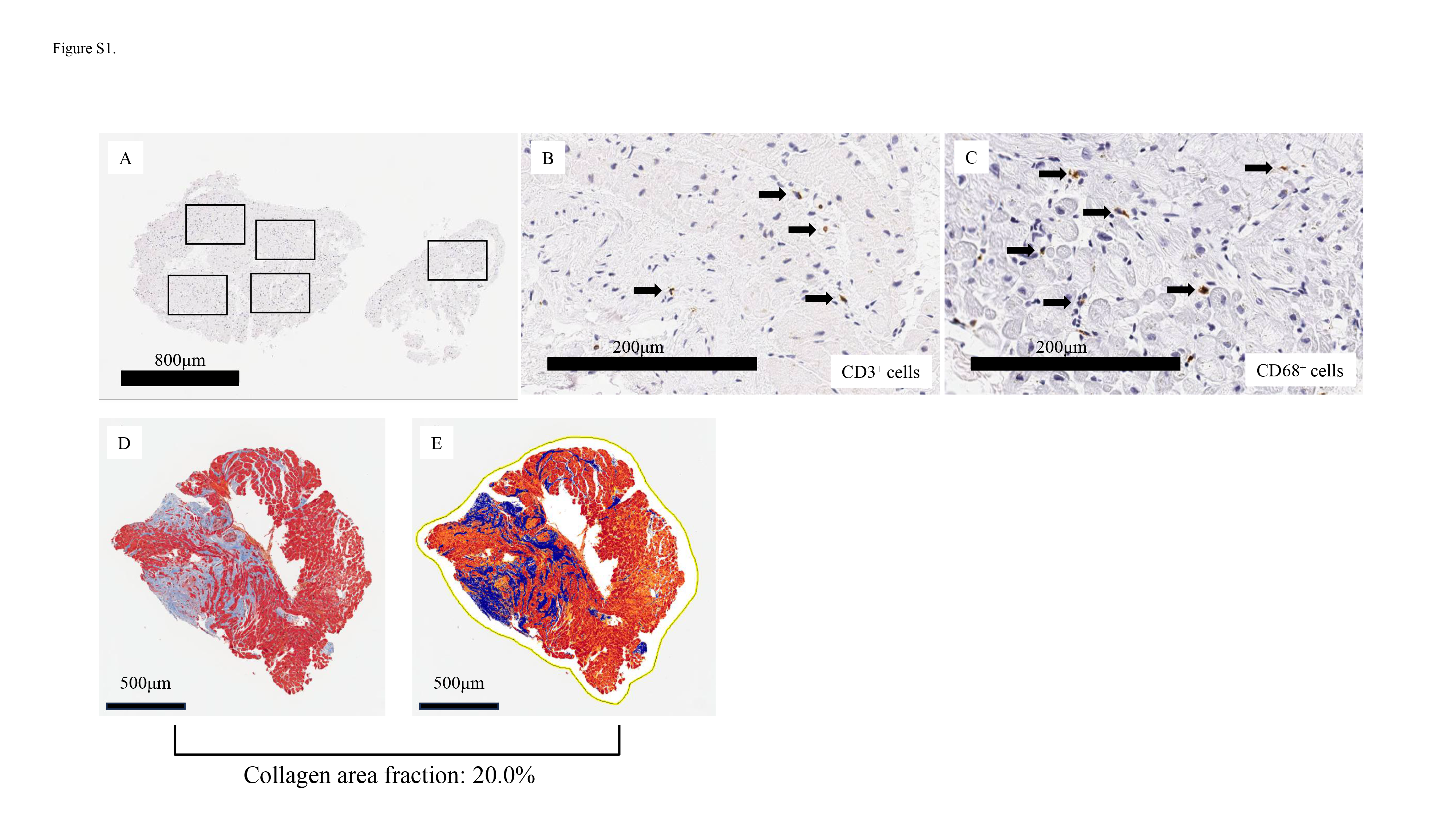

Supplement: online supplemental figure 1 [file openhrt-12-1-s001.tif]

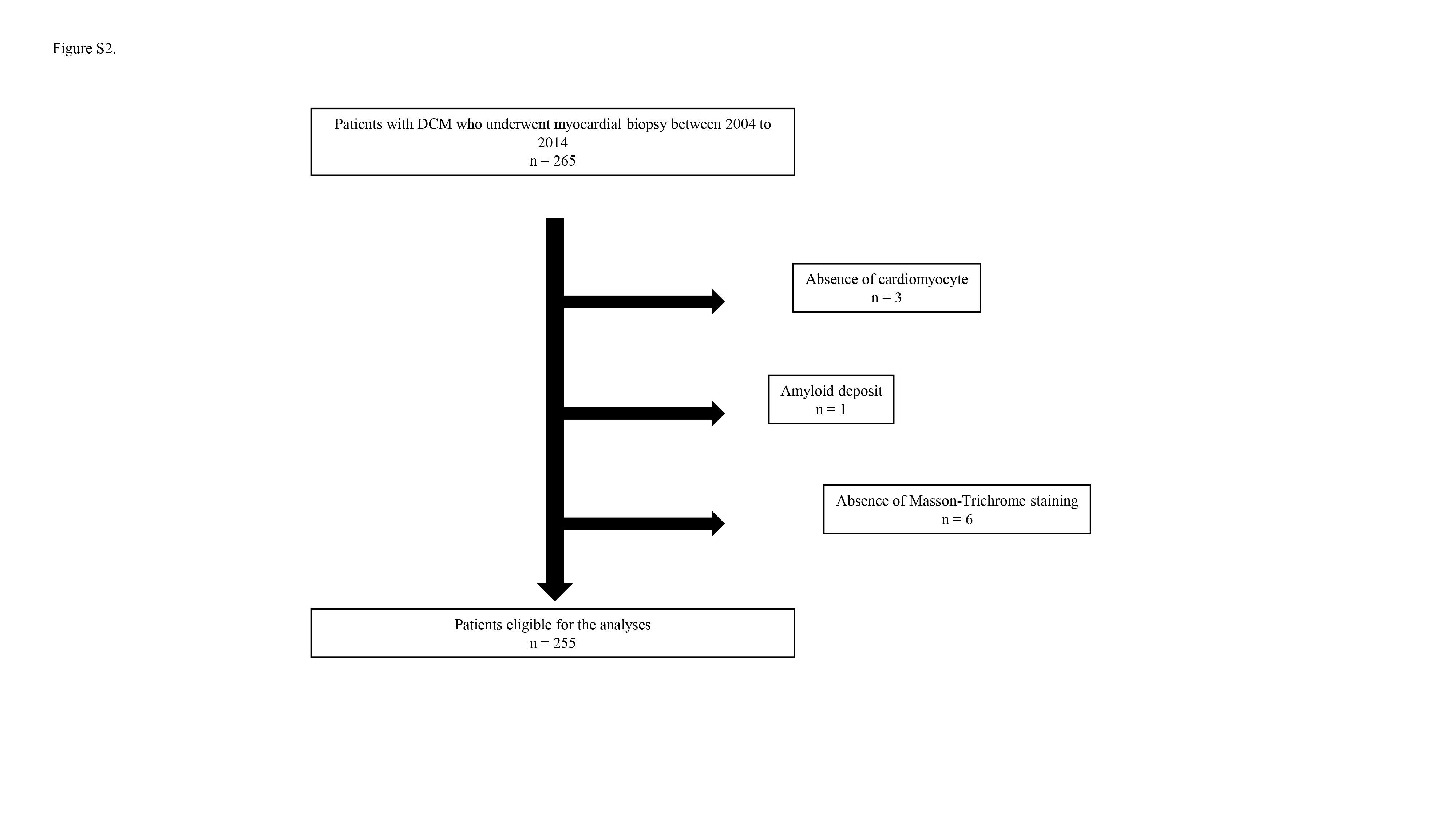

Supplement: online supplemental figure 2 [file openhrt-12-1-s002.tif]

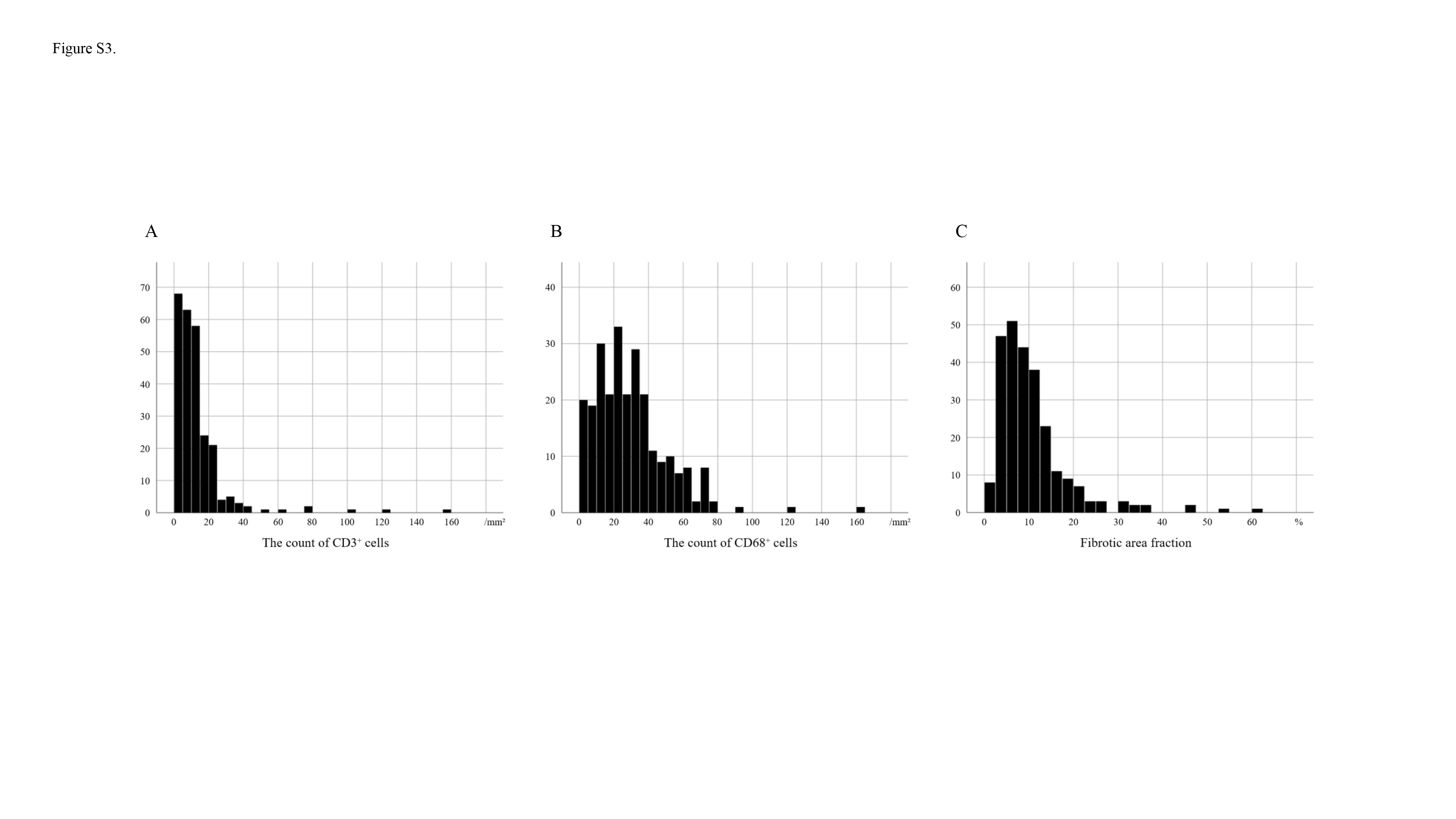

Supplement: online supplemental figure 3 [file openhrt-12-1-s003.tif]

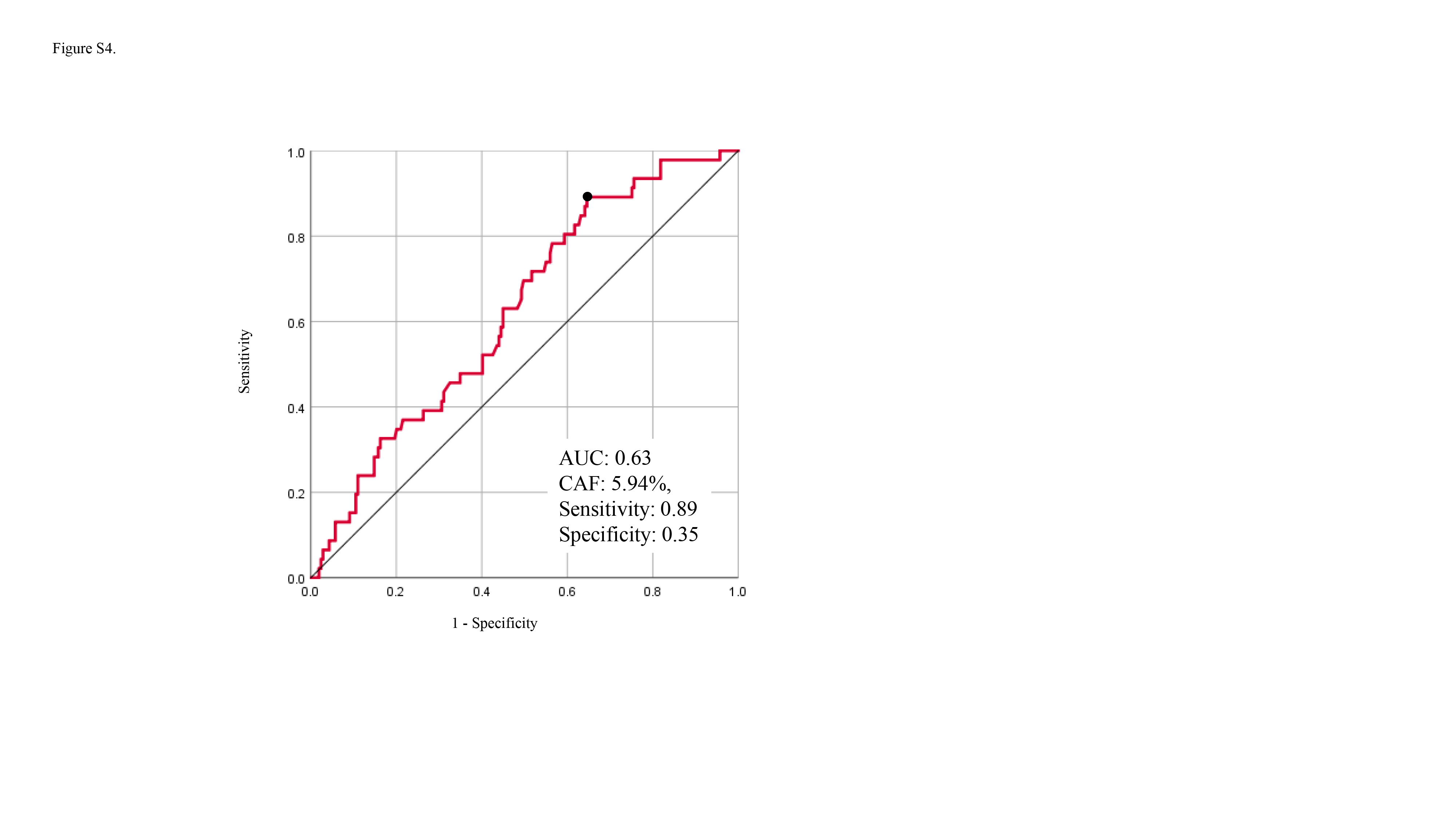

Supplement: online supplemental figure 4 [file openhrt-12-1-s004.tif]

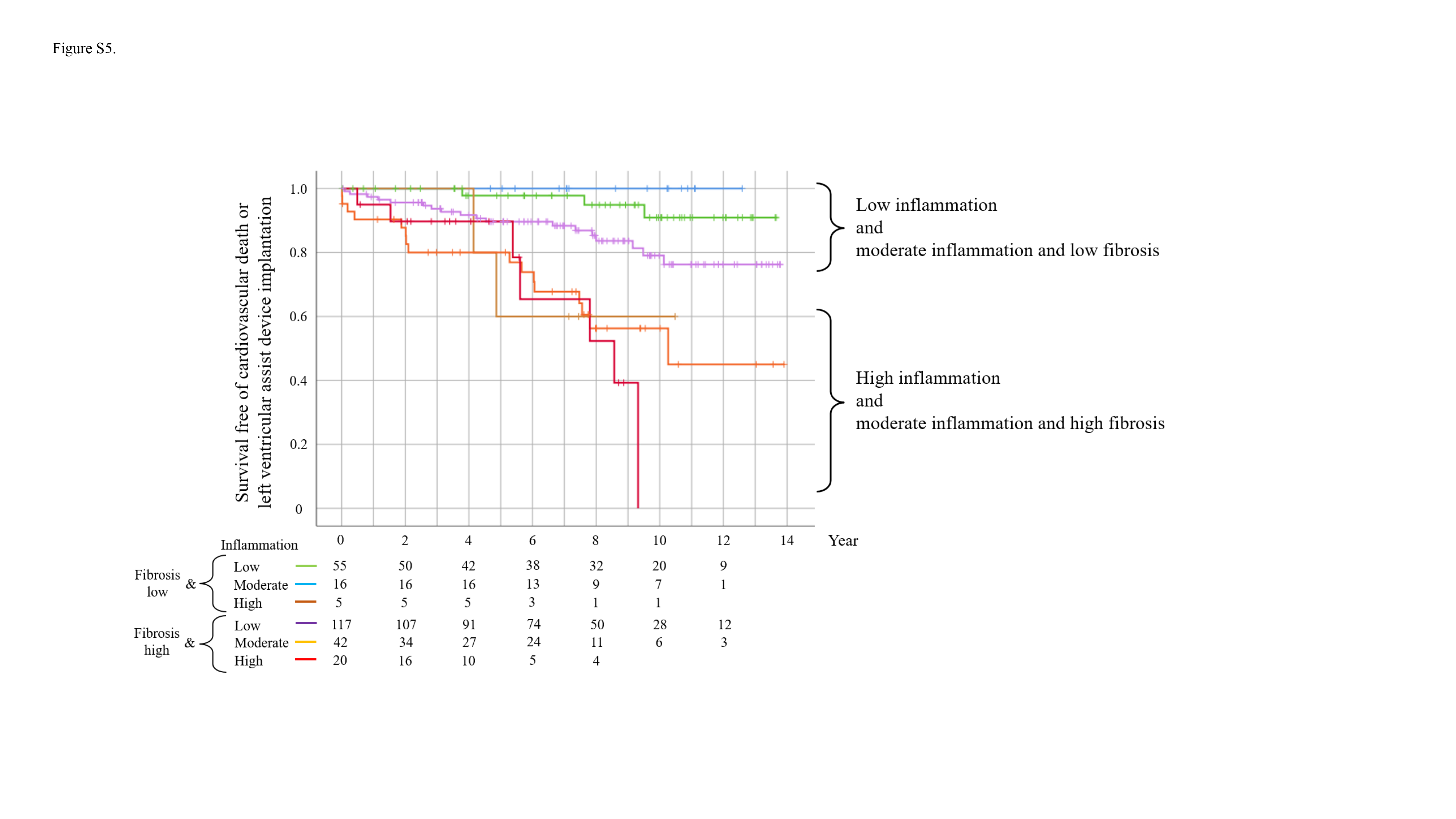

Supplement: online supplemental figure 5 [file openhrt-12-1-s005.tif]

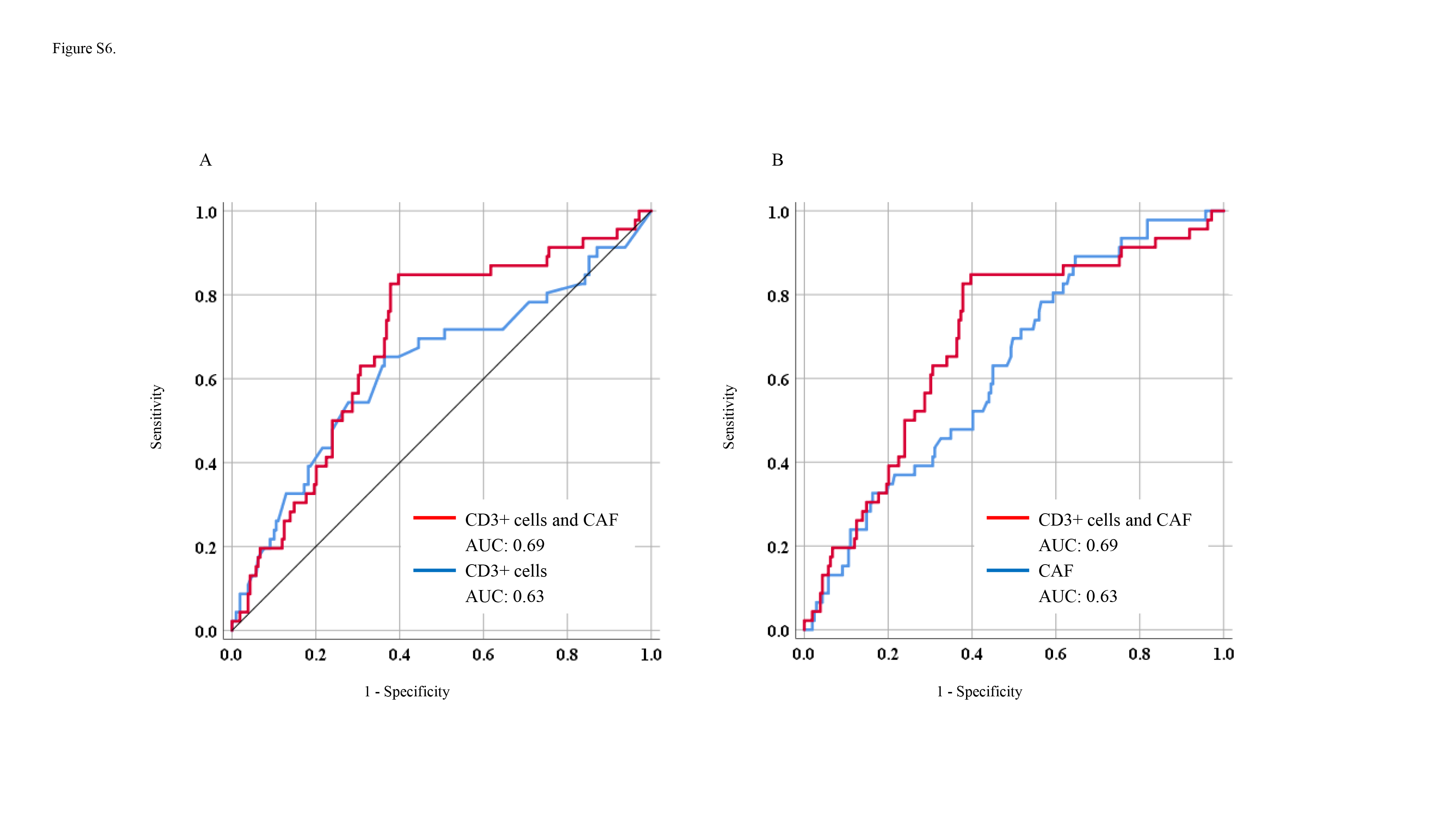

Supplement: online supplemental figure 6 [file openhrt-12-1-s006.tif]

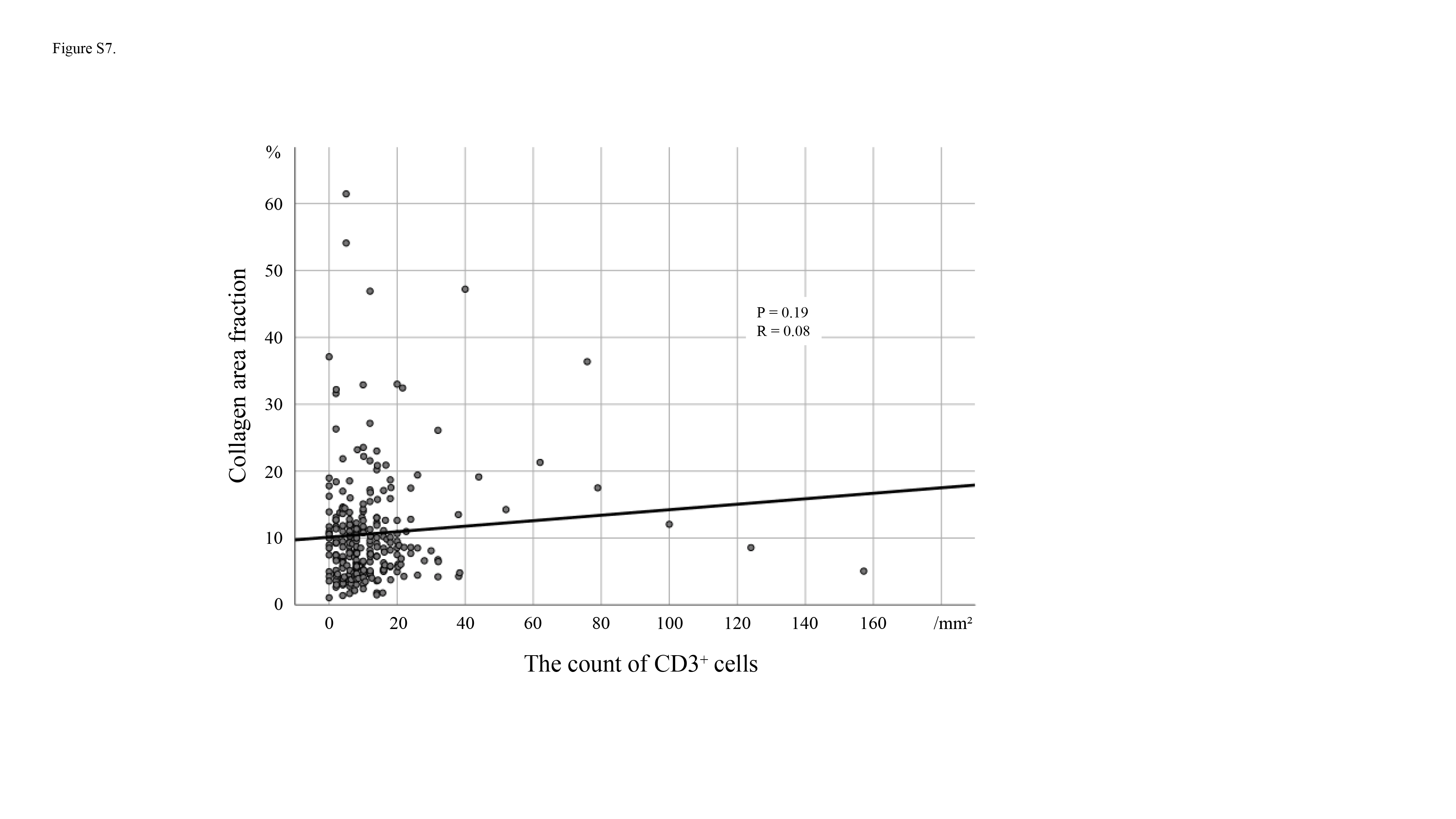

Supplement: online supplemental figure 7 [file openhrt-12-1-s007.tif]

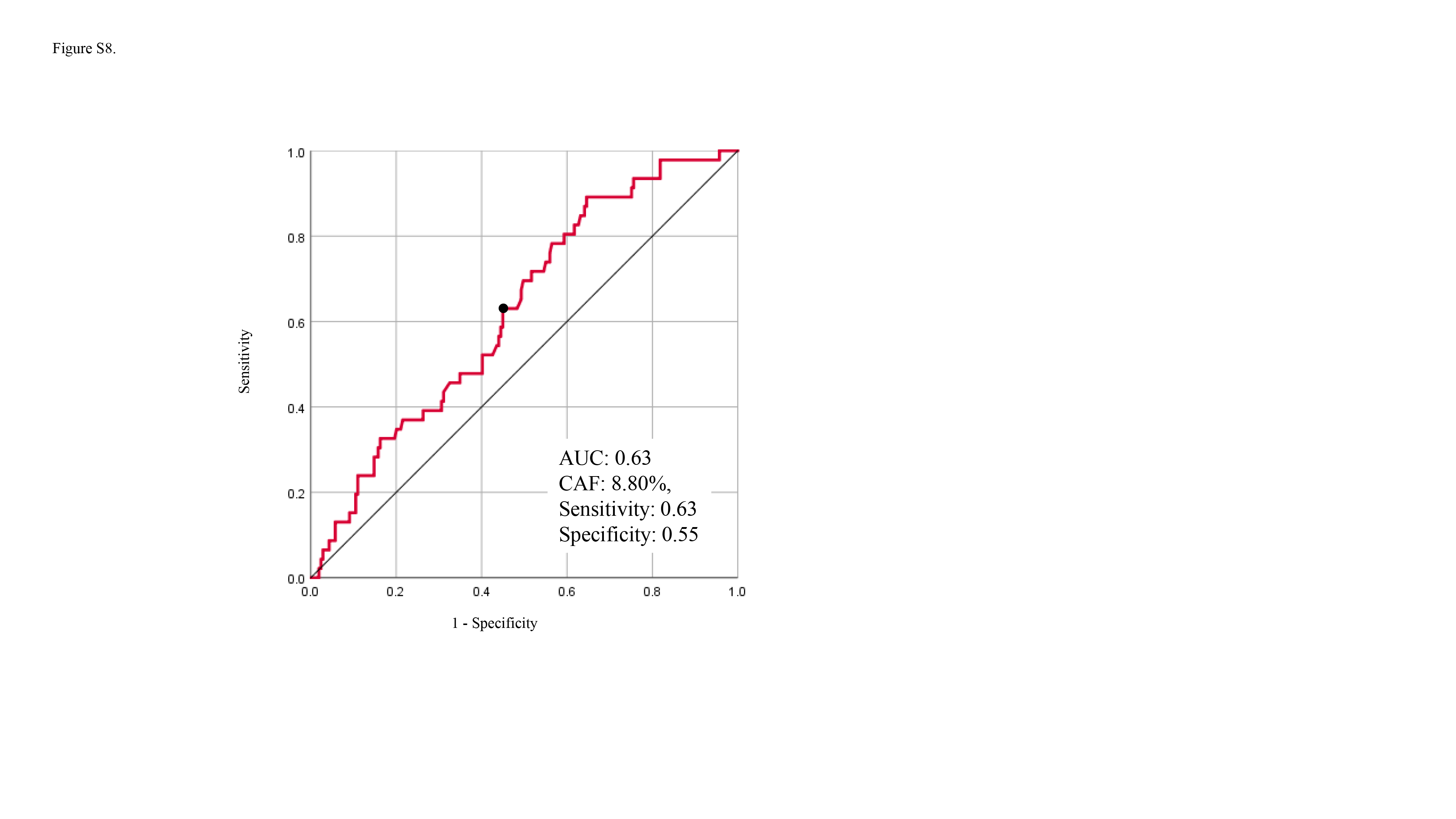

Supplement: online supplemental figure 8 [file openhrt-12-1-s008.tif]

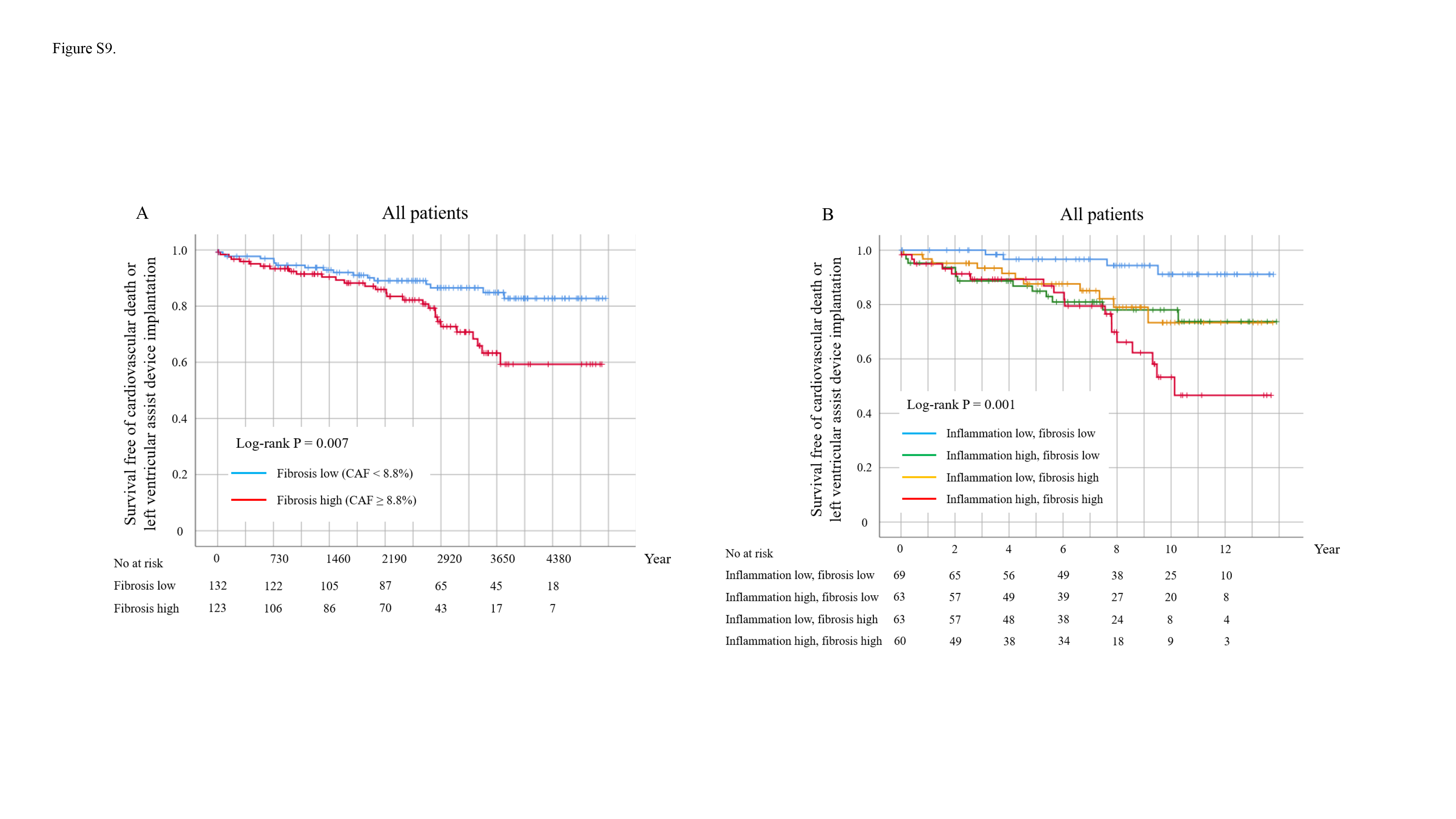

Supplement: online supplemental figure 9 [file openhrt-12-1-s009.tif]

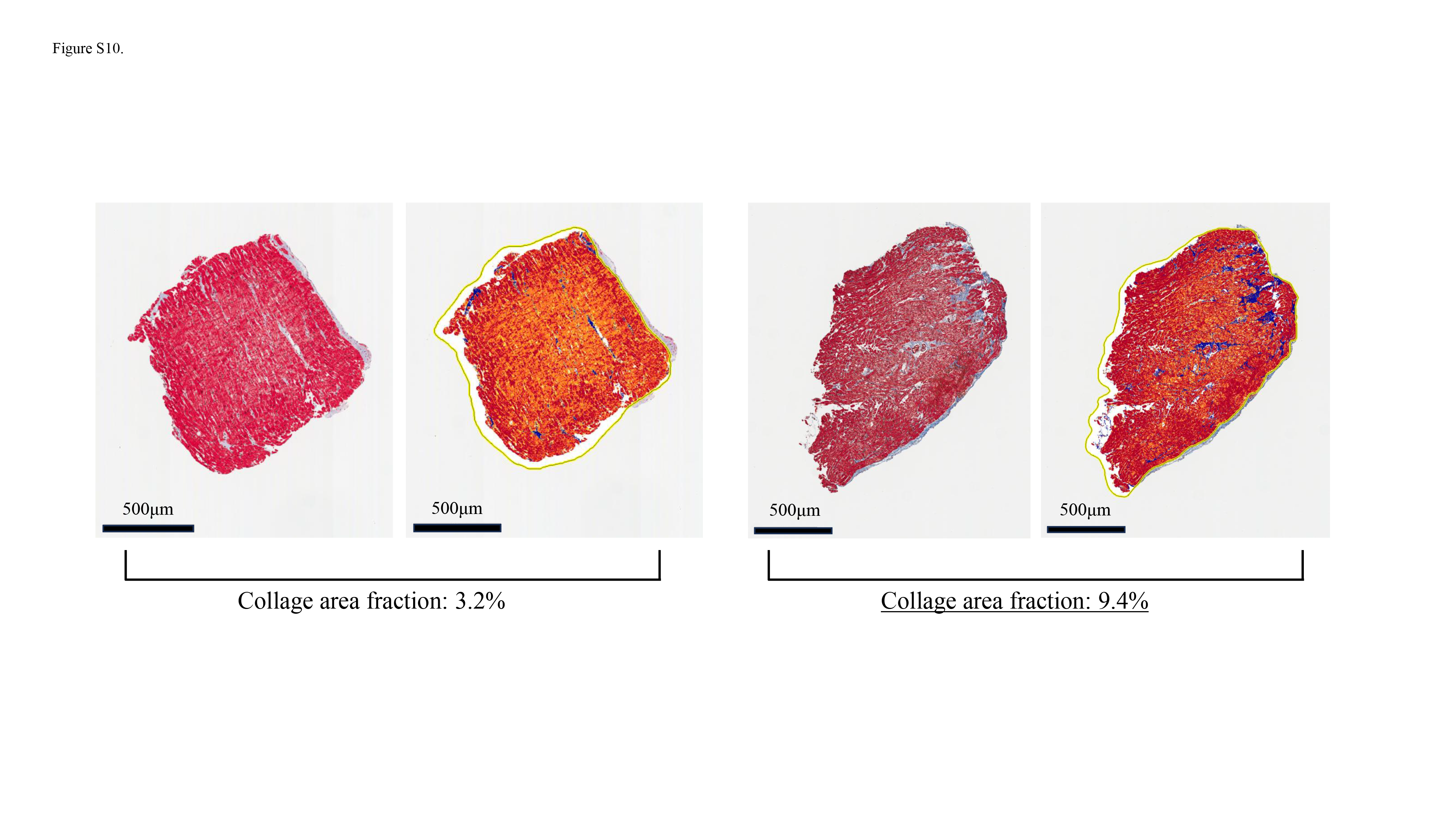

Supplement: online supplemental figure 10 [file openhrt-12-1-s010.tif]
