# Supplementary material for: Clinical impact of combined assessment of myocardial inflammation and fibrosis using myocardial biopsy in patients with dilated cardiomyopathy: a multicentre, retrospective cohort study
Source: Open Heart. 2025 Mar 12;12(1):e003250. doi: 10.1136/openhrt-2025-003250 (PMC11907087; doi:10.1136/openhrt-2025-003250)
Supplement: online supplemental table 1 [file openhrt-12-1-s011.docx]

| Table S1. Detailed products and methods |  |
| --- | --- |
| Autostainer | Leica Bond-3 (Leica Biosystems, Wetzlar, Germany) |
| Anti-CD3 antibody | N1580, 1:10 dilution; Dako, Glostrup, Denmark |
| Anti-CD68 antibody | PG-M1, N0876, 1:1000 dilution; Dako |
| Antibody detection and counter-staining | Bond Polymer Refine Detection kit (DS9800; Leica Biosystems) |
| Negative control to exclude false-positive from the secondary antibody or non-specific IgG binding | Mouse immunoglobulin G (IgG) 1 antibody (X0931; Dako) |
| Digital scanning | Aperio Scan-Scope XT (Leica Biosystems) |

| Table S2. Logistic regression analysis for myocardial inflammation (ESC criteria) | | | | | |  |  |
| --- | --- | --- | --- | --- | --- | --- | --- |
|  | Univariable | |  | Multivariable | | | |
|  | OR | P value |  | OR | P value | | |
| **Demographics** |  |  |  |  |  | | |
| Age, per 10-year | 0.82 (0.69-0.97) | 0.019 |  | 0.80 (0.68-0.95) | 0.012 | | |
| Male, vs. female | 1.51 (0.83-2.72) | 0.18 |  |  |  | | |
| Body mass index, kg/m^2^ | 1.00 (0.95-1.06) | 0.99 |  |  |  | | |
| NYHA class | 1.45 (1.09-1.93) | 0.010 |  | 1.49 (1.12-1.99) | 0.007 | | |
| Duration of hear failure,  per-5 month (n=173) | 1.02 (0.99-1.06) | 0.21 |  |  |  | | |
| Hypertension | 0.71 (0.43-1.17) | 0.18 |  |  |  | | |
| Dyslipidemia | 0.64 (0.39-1.05) | 0.079 |  |  |  | | |
| Diabetes mellitus | 0.81 (0.39-1.72) | 0.59 |  |  |  | | |
| Atrial fibrillation | 0.89 (0.50-1.57) | 0.69 |  |  |  | | |
| Smoking history (n=233) | 1.01 (0.60-1.72) | 0.96 |  |  |  | | |
| Chronic obstructive pulmonary disease | 0.26 (0.03-2.38) | 0.23 |  |  |  | | |
| **Laboratory measurements** |  |  |  |  |  | | |
| Log BNP | 1.27 (0.82-1.98) | 0.29 |  |  |  | | |
| Estimated GFR, per 10-mL/min/1,73m2 | 1.01 (0.89-1.14) | 0.94 |  |  |  | | |
| White blood cells, per 1,000 /µL | 1.02 (0.89-1.17) | 0.76 |  |  |  | | |
| Neutrophils, per 1,000 /µL (n=228) | 0.92 (0.77-1.10) | 0.36 |  |  |  | | |
| Lymphocytes, per 1,000 /µL (n=228) | 1.28 (0.85-1.91) | 0.24 |  |  |  | | |
| C-reactive protein, mg/dL | 0.71 (0.46-1.10) | 0.12 |  |  |  | | |
| **Echocardiography** |  |  |  |  |  | | |
| LVEDD, mm | 1.00 (0.97-1.03) | 0.86 |  |  |  | | |
| LVEF, per-10% | 0.82 (0.61-1.09) | 0.17 |  |  |  | | |
| **Hemodynamics** |  |  |  |  |  | | |
| PCWP, mmHg | 1.03 (1.00-1.06) | 0.095 |  |  |  | | |
| Cardiac index, L/min/m^2^ | 0.99 (0.77-1.26) | 0.91 |  |  |  | | |
| **Pathological findings** |  |  |  |  |  | | |
| Fibrosis area > 5.9 % | 1.22 (0.71-2.09) | 0.47 |  |  |  | | |
| Abbreviations: NYHA, New York Heart Association; BNP, brain natriuretic peptide; GFR, glomerular filtrating ratio; LVEDD, left ventricular end-diastolic diameter; LVEF, left ventricular ejection fraction; PCWP, pulmonary capillary wedged pressure. | | | | | |  |  |
|  |  |  |  |  |  |  |  |
|  |  |  |  |  |  |  |  |
|  |  |  |  |  |  |  |  |

| Table S3. Logistic regression analysis for myocardial fibrosis (> 5.9 %) | | |  |  | | |  | |  |  |
| --- | --- | --- | --- | --- | --- | --- | --- | --- | --- | --- |
|  | Univariable | | | |  | Multivariable | | | | |
|  | OR | P value | | |  | OR | | P value | | |
| **Demographics** |  |  | | |  |  | |  | | |
| Age, per 10-year | 0.93 (0.78-1.12) | 0.44 | | |  |  | |  | | |
| Male, vs. female | 0.81 (0.43-1.52) | 0.51 | | |  |  | |  | | |
| Body mass index, kg/m^2^ | 0.95 (0.90-1.02) | 0.14 | | |  |  | |  | | |
| NYHA class | 1.09 (0.80-1.47) | 0.59 | | |  |  | |  | | |
| Duration of hear failure,  per-5 month (n=173) | 1.04 (0.98-1.10) | 0.19 | | |  |  | |  | | |
| Hypertension | 0.49 (0.28-0.85) | 0.011 | | |  | 0.51 (0.29-0.92) | | 0.025 | | |
| Dyslipidemia | 0.86 (0.50-1.47) | 0.58 | | |  |  | |  | | |
| Diabetes mellites | 0.93 (0.42-2.06) | 0.85 | | |  |  | |  | | |
| Atrial fibrillation | 1.20 (0.64-2.27) | 0.57 | | |  |  | |  | | |
| Smoking history (n=233) | 1.14 (0.64-2.04) | 0.66 | | |  |  | |  | | |
| Chronic obstructive pulmonary disease | 0.10(0.01-0.92) | 0.042 | | |  | 0.12 (0.01-1.08) | | 0.058 | | |
| **Laboratory measurements** |  |  | | |  |  | |  | | |
| Log BNP | 2.13 (1.29-3.53) | 0.003 | | |  | 1.74 (0.97-3.13) | | 0.064 | | |
| Estimated GFR, per 10-mL/min/1,73m2 | 1.07 (0.95-1.19) | 0.26 | | |  |  | |  | | |
| White blood cells, per 1,000 /µL | 1.02 (0.88-1.19) | 0.77 | | |  |  | |  | | |
| Neutrophils, per 1,000 /µL (n=228) | 1.08 (0.8901.31) | 0.45 | | |  |  | |  | | |
| Lymphocytes, per 1,000 /µL (n=228) | 0.81 (0.52-1.25) | 0.34 | | |  |  | |  | | |
| C-reactive protein, mg/dL | 1.16 (0.74-1.83) | 0.52 | | |  |  | |  | | |
| **Echocardiography** |  |  | | |  |  | |  | | |
| LVEDD, mm | 0.99 (0.96-1.03) | 0.69 | | |  |  | |  | | |
| LVEF, per-10% | 0.86 (0.63-1.18) | 0.36 | | |  |  | |  | | |
| **Hemodynamics** |  |  | | |  |  | |  | | |
| PCWP, mmHg | 1.04 (1.00-1.08) | 0.037 | | |  | 1.02 (0.97-1.06) | | 0.48 | | |
| Cardiac index, L/min/m^2^ | 0.85 (0.63-1.13) | 0.25 | | |  |  | |  | | |
| **Pathological findings** |  |  | | |  |  | |  | | |
| Inflammation (ESC criteria) | 1.22 (0.71-2.09) | 0.47 | | |  |  | |  | | |
| Abbreviations: NYHA, New York Heart Association; BNP, brain natriuretic peptide; GFR, glomerular filtrating ratio; LVEDD, left ventricular end-diastolic diameter; LVEF, left ventricular ejection fraction; PCWP, pulmonary capillary wedged pressure. | | | | | | | | |  |  |
|  |  |  |  |  |  |  |  |  |  |  |
|  |  |  |  |  |  |  |  |  |  |  |
|  |  |  |  |  |  |  |  |  |  |  |

| Table S4. Cox proportional hazard analyses for the primary endpoint | | | | | |
| --- | --- | --- | --- | --- | --- |
|  | Univariable | |  | Multivariable | |
|  | HR | P value |  | HR | P value |
| **Laboratory measurements (systemic inflammation)** |  |  |  |  |  |
|  |  |  |  |  |  |
| White blood cells, per 1,000 /µL | 1.03 (0.88-1.21) | 0.72 |  | - | - |
| Neutrophils, per 1,000 /µL (n=228) | 1.09 (0.90-1.31) | 0.38 |  | - | - |
| Lymphocytes, per 1,000 /µL (n=228) | 0.54 (0.32-0.90) | 0.019 |  | 0.62 (0.38-1.03) | 0.064 |
| **Pathological findings** |  |  |  |  |  |
| CD3^+^ cells, per 5/mm^2^ | 1.10 (1.05-1.16) | < 0.001 |  | 1.10 (1.01-1.19) | 0.022 |
| Collagen area fraction, % | 1.04 (1.01-1.06) | 0.010 |  | 1.03 (1.00-1.06) | 0.046 |

| Table S5. The echocardiographic changes ("6-12 months after" - baseline) | | | |  |
| --- | --- | --- | --- | --- |
|  | ESC criteria positive and CAF > 5.9%, n = 71 | the other groups, n = 129 | P value |  |
| Δ LV diastolic diameter, mm | -3.0 (-10.0 - 0.0) | -9.0 (-13 - -3.0) | 0.001 |  |
| Δ LV systolic diameter, mm | -4.0 (-13.0 - 0.1) | -12.0 (-18.5 - -4.0) | < 0.001 |  |
| Δ LV ejection fraction, % | 8.9 (-1.1 - 18.0) | 11.3 (3.0 - 24.9) | 0.032 |  |
| Data are presented by the median (interquartile range) as the values are non-parametric. Abbreviations: ESC, European Society of Cardiology; CAF, collagen area fraction; LV, left ventricular. | | | |  |
|  |  |  |  |  |
|  |  |  |  |  |

| Table S6. Cox proportional hazard analyses for the primary endpoint | | | |  |  |
| --- | --- | --- | --- | --- | --- |
|  | Univariable | |  | Multivariable | |
|  | HR | P value |  | HR | P value |
| **Demographics** |  |  |  |  |  |
| Systolic blood pressure, per 5 mmHg (n = 77) | 0.82 (0.73-0.95) | 0.006 |  | 0.86 (0.75-0.99) | 0.039 |
| Hypertension | 0.33 (0.15-0.70) | 0.004 |  | 0.21 (0.03-1.70) | 0.14 |
| Multivariable analysis was performed on 77 patients. | | | | | |
|  |  |  |  |  |  |
|  | Univariable | |  | Multivariable | |
|  | HR | P value |  | HR | P value |
| **Demographics** |  |  |  |  |  |
| Systolic blood pressure, per 5 mmHg (n = 77) | 0.82 (0.73-0.95) | 0.006 |  | 0.79 (0.67-0.94) | 0.007 |
| Hypertension | 0.33 (0.15-0.70) | 0.004 |  | 0.36 (0.04-3.02) | 0.35 |
| **Pathological findings** |  |  |  |  |  |
| CD3^+^ cells, per 5/mm^2^ | 1.10 (1.05-1.16) | < 0.001 |  | 1.29 (1.04-1.60) | 0.020 |
| Collagen area fraction, % | 1.04 (1.01-1.06) | 0.010 |  | 1.08 (1.01-1.15) | 0.017 |
| Multivariable analysis was performed on 77 patients. | | | | | |
